# A rare case of pediatric recurrent rhabdomyolysis with compound heterogenous variants in the *LPIN1*

**DOI:** 10.1186/s12887-020-02134-5

**Published:** 2020-05-14

**Authors:** Ruochen Che, Chunli Wang, Bixia Zheng, Xuejuan Zhang, Guixia Ding, Fei Zhao, Zhanjun Jia, Aihua Zhang, Songming Huang, Quancheng Feng

**Affiliations:** 1 Department of Nephrology, Children’s Hospital of Nanjing Medical University, 72 Guangzhou Road, Nanjing, 210029 Jiangsu Province China; 2 Nanjing Key Laboratory of Pediatrics, Children’s Hospital of Nanjing Medical University, Nanjing, China; 3 Jiangsu Key Laboratory of Pediatrics, Nanjing Medical University, Nanjing, China

**Keywords:** Chinese, Genetic defect, *LPIN1*, Novel missense variant, Recurrent rhabdomyolysis, Case report

## Abstract

**Background:**

Lipin-1, encoded by *LPIN1* gene, serves as an enzyme and a transcriptional co-regulator to regulate lipid metabolism and mitochondrial respiratory chain. Autosomal recessive mutations in *LPIN1* were recognized as one of the most common causes of pediatric recurrent rhabdomyolysis in western countries. However, to date, there were only a few cases reported in Asian group. This study aims to report the first pediatric case of recurrent rhabdomyolysis with a novel *LPIN1* mutation in China mainland in order to raise the awareness of both pediatricians and patients.

**Case presentations:**

Here we report a Chinese pediatric case of recurrent rhabdomyolysis with compound heterozygous variants (p.Arg388* and p.Arg810Cys) in the *LPIN1* gene. The c.2428C > T was a novel missense variant involved Arg-to-Cys substitution at position 810 (p.Arg810Cys), located in the highly conserved region which predicted to be damaging by multiple algorithms. The patient manifested as cola-colored urine, muscle weakness and tenderness, as well as acute kidney injury with peak blood creatine kinase level 109,570 U/l in 19-month old. In his second episode of 9 years old, the symtoms were relatively milder with peak creatine kinase level 50,948 U/l. He enjoyed quite normal life between the bouts but slightly elevation of serum creatine kinase level during the fever or long-term exercises. Prolonged weight training combined with calorie deprivation were speculated to be the triggers of his illness. Prompt symptomatic therapy including fluid therapy and nutritional support was given and the patient recovered soon.

**Conclusions:**

*LPIN1*-related rhabdomyolysis is still quite new to physicians due to its seemly low-incidence especially in Asian countries. In the future, more active genetic test strategy and detailed prophylactic care education should be taken in patients with severe recurrent rhabdomyolysis, who are the high risk group of *LPIN1* genetic defects.

## Background

Acute rhabdomyolysis, a life-threatening disorder, was characterized by myalgia, muscle weakness, myoglobinuria, renal failure, and secondary injury to other organ systems. Although trauma, intoxication and infection were the major causes of rhabdomyolysis in adults, metabolic myopathies should be suspected when it comes to recurrent rhabdomyolysis especially in the childhood. In metabolic myopathies, mitochondrial diseases, lipid metabolism defect, glycolytic/glycogenolytic defect and some certain gene mutations (*RYR1*, *ALDOA* or *LPIN1*) were the major culprits [1]. *LPIN1* gene encodes lipin-1, which acts at the endoplasmic reticulum to dephosphorylate phosphatidic acid (PA) to form diacylglycerol (DAG), a precursor of lipid. In addition, lipin-1 also serves as a transcriptional co-regulator with some peroxisome proliferator-activated receptors (PPARs) to regulate lipid metabolism and mitochondrial respiratory chain [2]. When energy is sufficient, lipin-1 could be phosphorylated by the nutrient-activated mammalian target of rapamycin (mTOR) kinase and retented in the cytosol. Nevertheless, during nutrient shortage, it translocates to the nucleus, interacting with transcriptional factors or co-activators involved in metabolic gene expression. Primary myoblasts from lipin-1 deficient patients exhibited a dramatic decrease in *LPIN1* expression and PAP activity, associated with a significant accumulation of lipid droplets [3]. In 2008, Zeharia et al. first reported that autosomal recessive mutations in *LPIN1* cause recurrent acute myoglobinuria in childhood [4]. To date, more than 60 patients with 30 variants were reported in HGMD and literature. Mutations in *LPIN1* were gradually recognized as the one of the most common causes of recurrent rhabdomyolysis, as the incidence reported for patients suffering from severe rhabdomyolysis with onset before age 6 years and creatine kinase (CK) > 10,000 U/L reaches 46% by a series of studies [1, 5]. However, to date, there were only a few cases reported in Asia. Here we reported a 9-year-old boy suffered from recurrent rhabdomyolysis with compound heterogenous variants in *LPIN1*.

## Case presentation

A 9-year-old boy was admitted in the hospital because of ‘cola-colored urine for one day’. Before the day of the onset, the boy practiced throwing the 1 kg solid ball for 45 min. The solid ball is a rubber ball with sands inside, which is used for testing the strength and speed of students in parts of China. After going back home, he missed the supper and went to bed earlier than before. It is estimated that he had fasted for at least 12 h. The next morning, he complained of loin and the muscle of both lower extremities tenderness as well as passing dark-colored urine. There was no infection clues. The vital signs were stable. Serum biochemical tests were shown in Table 1. Urinalysis showed mild proteinuria without any red blood cells. Urine dipstick was positive for myoglobin and hemoglobin. Rhabdomyolysis was diagnosed. Past history indicated that he had similar but more severe rhabdomyolysis bout when he was 19-month-old. At that time, he ate several river prawns and then became lethargy, weak and oliguria. He was also reported a history of upper respiratory tract infection by his parents at that time. His urine turned into dark cola-colored. After admitting to the intensive care unit, he had fever, mild cough, muscle pain and hypotonia in both lower extremities. Biochemical results were also shown in Table 1. His serum creatinine increased to 101.2 umol/l, more than 3 times baseline (21.5 umol/l after his recovery). He got full recovery after 15 days, supported by consistent renal replacement therapy (CRRT) for 5 days and proper fluid therapy. The CK concentration remains normal between the episodes while he still got mild CK elevation when he was 4 and 7 years old. The possible triggers include long-term exercises and fever. The parents claimed the boy had done blood acyl-carnitine profiling using tandem mass spectrometry before and the results were negative but we didn’t see the reports. His motor and cognitive development were uneventful. He has neither any other chronic diseases nor long-term medicine taken. In the daily life, he could tolerate swimming and jogging. There was no family history of rhabdomyolysis or any other musculoskeletal diseases. The parents were both Chinese and non-consanguineous. Supportive therapies including taking full rest, fluid therapy and nutrition support were given and the CK, ALT, AST and LDH had gradually improved. His urine turned clear. Repeated urine myoglobin and hemoglobin testing were negative.

**Table 1:** Biochemical findings of the patient

Serum Biochemistry	2011–06	2019–10
ALT (U/l)	1130	157
AST (U/l)	2790	829
LDH (U/l)	8000	820
CK (U/l)	109,570	50,948
CK-MB (U/l)	1460	902
Myoglobin (ng/ml)	> 4057	> 4057
cTnI (ng/ml)	< 0.2	< 0.2
Cr (umol/l)	101.2	30
BUN (mmol/l)	18.9	3
Cholesterol (mmol/l)	2.82	3.89
Triglycerides (mmol/l)	1.84	0.88

Table legend: The table represented the peak value of serum biochemical findings in two bouts of recurrent rhabdomyolysis (2011–06, 2019–10). Abbreviations: *ALT* Alanine aminotransferase, *AST* Aspartate transaminas, *LDH* Lactate dehydrogenase, *CK* Creatine kinase, *CK-MB* Creatine kinase myocardia band, *cTnI* Cardiac troponin I, *Cr* Creatinine, *BUN* Blood urea nitrogen

The parents refused to do the muscle biopsy and electromyogram (EMG) but accepted the whole exome sequencing (WES). The method of WES was mentioned in our previous report [6]. To verify the mutated point, we amplified the segments carrying the mutations. Two primer pairs ‘forward: TGCCCAAACCTCAAAGTATTTGTC, reverse: AACATAAGGGGAAACTGGTCTCA’ and ‘forward: GTCTGAACAGGCTCACAGATGG, reverse: AGGATTAAACAAAGTGGGGAATGC’ were designed, respectively. The PCR mixture, a total volume of 25 μl, contained 2.0 μl of DNA, 1.5 μl of primers, 12.5 μl of 2 × Taq Master Mix (Vazyme Biotech Co., Ltd) and 9 μl of ddH2O. Cycling conditions included a predenaturation step at 94 °C for 5 min, followed by 34 cycles at 94 °C for 30s, 59 °C for 30s and 72°Cfor 30s, with a final extension at 72 °C for 5 min.. The purified PCR products were sequenced by 3730XL DNA Analyzer (Applied Biosystems). *LPIN1* gene variant (GenBank association number NM_145693) was utilized as a reference sequence.

**Fig. 1:**
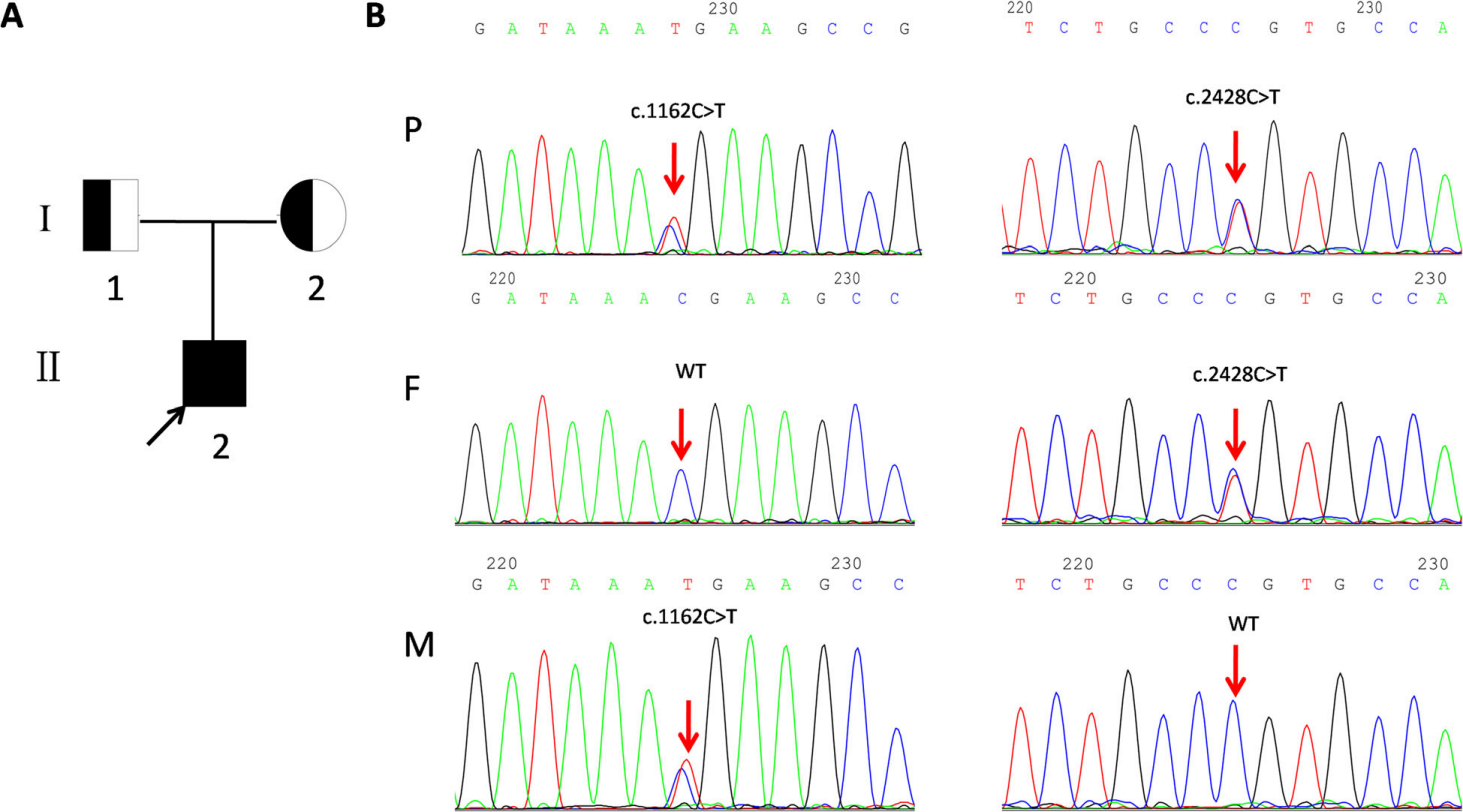
Pedigree of the Chinese family with two different *LPIN1* mutations. A: proband (II-1) is shown. The squares represent the proband, and his father. The circle represent the mother. B: direct sequencing showing two alleles of the proband and his parents, respectively. P: proband, F: father, M: mother

By WES and subsequent direct sequencing of the *LPIN1* gene, we identified the boy carried heterozygous compound variants c.1162C > T (p.Arg388*); c.2428C > T(p.Arg810Cys) which were inherited from his mother and father, respectively (Fig. 1). The c.1162C > T was a known nonsense variant which creates a stop Condon at residue 388 (p.Arg388*), were reported to result in truncated proteins lacking catalytic activity either in homozygous or compound heterozygous mutations [1, 4, 7]. The c.2428C > T was a novel missense variant involved Arg-to-Cys substitution at position 810 (p.Arg810Cys), was classified as “likely pathogenic” (PM2 + PP3 + PP4 + PM3) according to American college of medical genetics and genomics (ACMG) guidelines [8]. This variant has not been reported in the genomic databases or the literature at the time of query. Moreover, the missense variant was absent in population databases (1000G [9] and gnomAD [10]). Various bioinformatic tools (SIFT, PolyPhen-2, Mutationtaster, REVEL and GERP) revealed scores associated with likely pathogenic effects for the missense variant. The p.Arg810Cys located in the highly conserved region which predicted to be damaging by multiple algorithms. Taken together, we believe that the compound heterozygous variants (p.Arg388* and p.Arg810Cys) are the molecular basis of this proband. Informed consent was sought from the patients for using the history and laboratory values for reporting purpose.

## Discussion and conclusions

In this study, we identified compound heterozygous variants (p.Arg388* and p.Arg810Cys) in the *LPIN1* gene in a rare pediatric case with recurrent rhabdomyolysis. The proband suffered from recurrent rhabdomolysis especially in the catabolism status but presented quite normal in the daily life. We report the first case in Chinese mainland and the second case with Chinese ethnicity in English literature while Yim et al. shared the first Hong Kong Chinese case of acute recurrent rhabdomyolysis in a boy with compound heterozygous *LPIN1* variants [11].

**Fig. 2:**
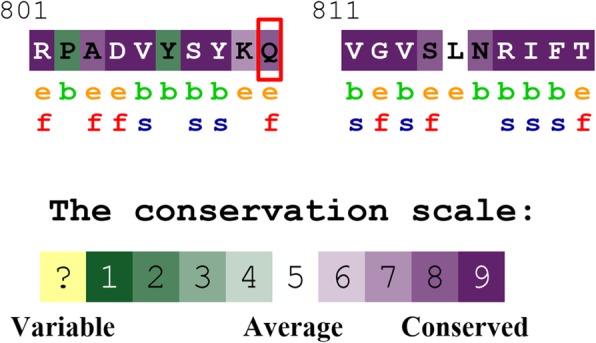
Evolutionary-conservation scores for residues encompassing the Q810 mutation from different species including mammals, lower vertebrates, invertebrates and lower eukaryotes. The evolutionary-conservation scores were obtained from the Uniref90 database. The results show that the Q residue (outlined by the red box) is highly conserved

The *LPIN1* gene, 149,865 bases length, 20 exons, is mapped to chromosome 2p25.1. Lipin-1, encoded by *LPIN1* gene, is Mg^2+^-dependent phosphatidate phosphatase enzyme that consists of 890 amino acids with two conserved domains, an N-terminal lipin (N-LIP) region that spans the first 108 residues, and the C-terminal lipin (C-LIP) domain at amino acids 624–830. The C-LIP region contains two critical domains, one is a transcriptional binding motif, Leu-Xaa-Xaa-Ile-Leu (LXXIL (678–682)), which mediates interaction with PPAR-α. Another is Asp-Xaa-Asp-Xaa-Thr (DXDXT (689–693)) motif, a catalytic motif essential for phosphatidate phosphatase activity. The position of Arg810 we found is highly conserved in orthologous lipin proteins from plants to humans (Fig. 2). It is in the vicinity of Asp804, a Mg^2+^-dependent catalytic site responsible for the PAP activity [12]. The novel missense mutation of c.2428C > T (p.Arg810Cys) might induce the loss of catalytic activity of lipin-1. The variant c.1162C > T, which converts Arg388 to a stop codon and truncates the enzyme by 502 residues, has been reported in several articles. There were 8 cases carrying the homozygous mutation [1, 4, 5, 13, 14] and 3 cases with compound heterzygous variants [1, 5, 7]. Interestingly, the clinical symptoms of homozygous mutation with c.1162C > T (p.Arg388*) varied from milder elevation of CK (16,000 U/L) with only one episode to severe increased CK (300,000 U/L) and several bouts [14], showing no definite genotype-phenotype correlation. But we still could find some clues. The frameshift mutations may lead to complete loss of protein expression or generation of severely truncated proteins lacking the carboxyl terminus. For example, a case of lipin-1 deficiency caused by uniparental isodisomy of maternal chromosome 2 with a homozygous frameshift mutation (c.1381delC) suffered from very high CK levels that diminished slowly and incompletely, unlike most of the cases. The complete loss of lipin-1 protein function is believed to be related with the severity of his rhabdomyolytic episodes [15]. In other ways, some mutations, like single amino acid substitutions, will cause milder defects with losing intrinsic phosphatidic acid phosphohydrolase activity while retaining transcriptional regulatory function [16, 17].

Although lipin-1 plays a critical role in the synthesis of phospholipids and triacylglycerol, the blood levels of metabolic components in our case, like triglycerides, creatinine, and cholesterol, remains normal, similar to the previous reports. Instead, in the presence of pro-inflammatory cytokines, acetyl-CoA carboxylase beta, a key enzyme in the fatty acid synthesis/oxidation balance was overexpressed in patients’ myotubes and resulted in the free fatty acid accumulation in patients’ myoblasts [3]. Meanwhile, the study by Legendre showed decreased ATP levels and oxidative phosphorylation in the myoblasts and skeletal muscle [14]. Therefore, it is speculated that lipin-1 defect should be involved in higher level of metabolic stress especially during crisis rather than major glucose or lipid metabolism process in normal condition. Rashid et al. observed increased sarcoplasmic reticulum stress which induced mitochondrial dysfunction in skeletal muscle-specific *LPIN1* knock-out mice. Treatment with the chaperone TUDCA and the fatty acid oxidation activator bezafibrate could improve muscle histology and strength of *LPIN1* mutants [18].

*LPIN1* mutations were considered as the common cause of recurrent rhabdomyolysis in western countries, but the incidence in Asian group was surprisingly low. On one hand, a deletion mutation spanning exon 18 has been noted in 86% of Caucasian patients, which is believed to be a founder effect [1]. On the other hand, the possibility of low recognition of the *LPIN1* mutation in Asian countries could not be excluded. Most cases of recurrent rhabdomyolysis enjoy a normal life in the episodes, which makes the diagnosis delayed and ambiguous. Actually, it always takes years to get full diagnosis not just in our case but also in most literature reports. According to the study by Michot, severe rhabdomyolysis bouts with onset before age 6 years and CK > 10,000 U/L was the highly risky group while cases with mild rhabdomyolysis (CK < 10,000 UI/L) or myalgias without rhabdomyolysis were not involved [5]. It is crucial to arouse the awareness of physicians to screen out the potential genetic defects in high risk group.

In our case, the intake of river prawns could not be ruled out as the trigger of first episode. Haff Disease, a rare syndrome of unexplained rhabdomyolysis following consumption of certain types of aquatic products, needs to be considered. Haff Disease was firstly reported in 1924 near the Königsberger Haff shores along the Baltic coast and to date, more than 1100 cases were reported in the whole world [19]. The mechanism underlying the disease is still unknown. But then the boy also got infectious clues of fever and cough, extremely high level of CK, liver and kidney function injury, with a pretty early onset, which was not in accord with Haff Disease [20]. Between episodes, the patient still occasionally participated in some physical exercises, like swimming and jogging, without any symptoms. Several clinical and basic research indicated that in prolonged exerices, cytokines could be secreted by skeletal muscle increasingly, which might be the a second strike to the myolysis*.* However, cytokines would not be dosed during efforts if the plasma lactate levels remained normal in *LPIN1*-deficiency patients without infection. In another word, short high intensity exercise would be tolerable in the absence of fever [14, 21, 22]. Another study from Austria suggested maintenance of caloric and water intake could alleviate the severity of rhabdomyolysis [23]. Therefore, we speculated prolonged weight training combined with calorie deprivation contributed to the multi-strike on our case. Medical advice was given including keep enough calorie and fluid intake, avoid triggers like statins administration, prolonged intensified muscle training, infection and fasting. In case of any anesthesia needed in the future possible surgeries, perioperative prophylactic administration of sufficient calorie and fluid as well as CK level surveillance was advised [24]. Since the evidence of the pathogical variants were strong enough, we did not perform the further transcriptional functional tests. That’s the limitation of the study.

In conclusion, *LPIN1*-related rhabdomyolysis was one of the major culprits in early-onset rhabdomyolysis, but it’s still quite new to physicians due to its seemly low-incidence especially in Asian countries. Considering its high mortality and ambiguous prognosis, it is necessary to draw the attention of both pediatric neurologists, cardiologist and nephrologists, the doctors most probably dealing with the cases. As soon as there is a risk factor, and of course as soon as there is a symptom, hospitalize the patient to hydrate as soon as possible and prepare for severe rhabdomyolysis with possible heart rhythm disorders and kidney impairment.

## Data Availability

The datasets used and/or analysed during the current study are available from the corresponding author on reasonable request.

## Abbreviations

ACMG: American college of medical genetics and genomics
ALT: Alanine aminotransferase
AST: Aspartate transaminase
BUN: Blood urea nitrogen
CK: Creatine kinase
CK-MB: Creatine kinase myocardia band
Cr: Creatinine
cTnI: Cardiac troponin I
C-LIP: C-terminal lipin
DAG: Diacylglycerol (DAG)
EMG: Electromyogram
LDH: Lactate dehydrogenase
mTOR: Mammalian target of rapamycin
N-LIP: N-terminal lipin
PA: Phosphatidic acid
PPARs: Peroxisome proliferator-activated receptors
WES: Whole exome sequencing
